# Formation and behavior of negative ions in low pressure aniline-containing RF plasmas

**DOI:** 10.1038/s41598-019-47425-9

**Published:** 2019-07-26

**Authors:** Cedric Pattyn, Eva Kovacevic, Thomas Strunskus, Thomas Lecas, Johannes Berndt

**Affiliations:** 1 GREMI Université d’Orléans, Polytech’Orleans, PB 6744, 45067 Orleans, Cedex 2 France; 2 Lehrstuhl für Materialverbunde, Technische Fakultät, Christian-Albrechts-Universität zu Kiel, Kaiserstraße 2, 24143 Kiel, Germany

**Keywords:** Plasma physics, Materials for devices

## Abstract

This paper is focused on the formation mechanisms and the general behavior of negative ions in low pressure radio-frequency (RF) plasmas operated in a mixture of argon and aniline vapor. This type of plasma is mostly used for the synthesis of polyaniline, one of the most studied conductive polymers. Experiments based on mass spectroscopy measurements reveal the necessity to have a thin layer of plasma synthesized polyaniline on the electrodes to produce negative ions through complex surface reactions. In addition, thin-films deposited using this type of discharge are analyzed by means of Near Edge X-ray Absorption Fine Structure spectroscopy (NEXAFS). The material analysis gives a first indication about the possible contribution of negative ions to polyaniline deposition.

## Introduction

Polyaniline (PANI) is a conductive polymer which has been widely studied for the last decades. Due to its high electrical conductivity and its chemical stability, this material has a great potential for various applications such as electrodes for organic LEDs^1–3^, supercapacitors^4–6^, transistors^7,8^, batteries^9,10^, or antistatic coatings for electronic packaging^11,12^. More recent works also highlighted the potential applications of polyaniline when it is structured at the nanometer scale, for example PANI nanoparticles, for environmental applications (dye removal)^13^, biological applications (cancer cell therapy and neurodegenerative diseases)^14^, and for the development of novel devices such as mercury ion sensors^15,16^.

**Figure 1:**
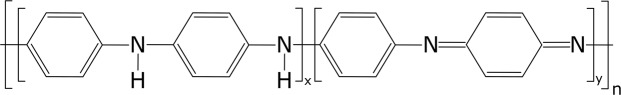
Scheme of the polyaniline structure. For x = y = 0.5, polyaniline has the emeraldine base structure. For x = 0 and y = 1, the structure is attributed to pernigraniline base and for x = 1 and y = 0, it is attributed to leucoemeraldine base.

Several methods are currently used for the synthesis of PANI. The most common one is the chemical oxidative polymerization of aniline which can be used to obtain different types of supramolecular PANI structures such as nanofibers, nanorods, nanoribbons, nanobelts or nanoparticles^17–19^. Another method involves the use of reactive plasmas (allowing the fast and solvent-free synthesis of this polymer), and is mostly used for the production of PANI thin-films and more recently PANI nanoparticles^20^. In addition to the benefits for the environment (solvent-free process), the use of plasmas is an attractive way to synthesize polyaniline, because of the capability of plasmas to produce dense and mostly pinhole-free polymers, with a precise control over the process (as e.g. the coating thickness or the nanoparticle radius). Polyaniline or polyaniline-like deposits have been synthesized by several plasma types ranging from capacitively and inductively coupled RF discharges, to ERC plasmas and DC glow discharges, to mention just a few of many articles^21–30^. While the materials have been extensively analyzed by different methods (XPS, FTIR, UV–Visible spectroscopy), and have been related in some of the works to certain plasma parameters^22,25,30,31^, examples for detailed plasma diagnostic studies are relatively rare in the literature^32,33^. A better understanding of the underlying plasma physical/chemical mechanisms is however essential in order to improve the deposition process and to guarantee a sufficient control over the structure of the synthesized material. This is particularly important considering the rather complex structure of PANI. PANI is known to exist in three different forms (leucoemeraldine base, pernigraniline base and emeraldine base) which represent three idealized oxidation states (see Fig. 1). The conductivity and stability of PANI is therefore highly dependent on its inherent structure, with the emeraldine base being the most stable and conductive form of PANI. A doping procedure is nevertheless necessary in order to turn emeraldine base into the highly conductive emeraldine salt.

In this paper we will mainly focus on the formation of negative ions in a capacitively coupled RF plasma operated in a mixture of argon and aniline vapor. It is well known that negative ions can be produced in low pressure plasmas used for Plasma Enhanced Chemical Vapor Deposition (PECVD), for example in oxygen (O^−^, O_2_^−^ and O_3_^−^)^34–38^, hydrogen (H^−^ or D^−^)^39–41^, silane (Si_n_^−^, Si_x_H_y_^−^…)^42–45^, hydrocarbon (C_n_^−^, C_x_H_y_^−^…)^46–48^, fluorocarbon (F_n_^−^, C_x_F_y_^−^…)^49–52^, or more generally in halogen plasmas (Cl^−^, Cl_2_^−^, F^−^, S_x_F_y_^−^…)^53–57^. Negative ions can strongly affect the behavior of a discharge by impacting for example the transport properties or the electron density and temperature^58–66^. Their influence on the nucleation of nanoparticles in low pressure reactive plasmas has moreover been highlighted in previous publications^47,67,68^. The study of negative ions in aniline plasmas is therefore an important point in the understanding of plasma based synthesis of polyaniline. This contribution mainly deals with mass spectroscopic measurements of negative ions produced in a low pressure capacitively coupled RF plasma (f = 13.56 MHz) ignited in a mixture of aniline vapor and argon and the influence of these ions on the deposition process. Mass spectra of both positive and negative ions are presented, the identification of the species created and the mechanisms of their formation are discussed. In addition, X-ray material analysis (Near Edge X-ray Absorption Fine Structure spectroscopy (NEXAFS), using a synchrotron radiation source) of plasma synthesized polyaniline (pPANI) are presented in correlation to negative ions behavior, in order to give a first indication of their contribution to thin-film growth.

## Results and Discussion

### Mass spectra of positive and negative ions

**Figure 2:**
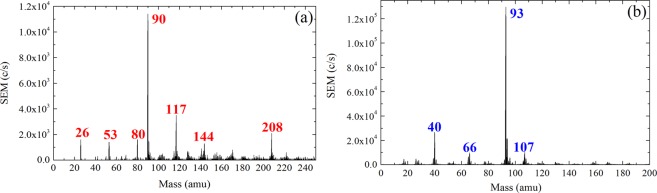
Mass spectrum of negative ions (**a**) and positive ions (**b**). Both measurements were performed from a pulsed plasma (pulsing frequency = 200 Hz) with a pressure of 0.18 mbar (argon: aniline = 20: 1 sccm) and with 5 W of input power.

Due to the confinement of negative ions in the positive plasma potential, the measurements were performed with pulsed discharges^20,47,58^. The negative ions could thus leave the discharge during the plasma off time and be detected by the mass spectrometer. Figure 2(a) shows a typical mass spectrum of negative ions found in this type of plasma. For pressures ranging from 0.1 to 0.3 mbar, and for different pulsing frequencies ranging from 25 Hz to 1200 Hz (no negative ions could be measured above 1200 Hz), the most abundant negative ions were systematically found for m/z = 90 amu, which is 3 mass units less than the aniline molecule (93.13 amu). The formula of the corresponding ion can be either C_6_H_4_N^−^ (formed by a multi-step mechanism from the aniline molecule) or C_7_H_6_^−^. Other negative species were measured for mass to charge ratios below the molecular mass of aniline, as e.g. for m/z = 26 amu (C_2_H_2_^−^ or CN^−^), 53 amu (C_4_H_5_^−^ or C_3_H_3_N^−^) and 80 amu (C_3_H_2_N_3_^−^, C_4_H_4_N_2_^−^, C_5_H_6_N^−^ or C_6_H_8_^−^), and above it as e.g. for m/z = 117 amu (most probably C_7_H_5_N_2_^−^, C_8_H_7_N^−^ or C_9_H_9_^−^), 144 amu and 208 amu. The molecular formula of the heavier species (m/z = 144 and 208 amu) is more difficult to discuss because of the numerous possibilities. Nevertheless the high intensity of negative ions signal for m/z = 208 amu which reaches about 20% of the absolute intensity measured for the dominant negative ions (m/z = 90 amu) indicates an important production of heavy negative species via volume polymerization in this type of discharge (especially considering the decrease of the mass spectrometer sensitivity for higher masses).

For positive ions (Fig. 2(b)), the two dominant species were systematically found at m/z = 93 amu (C_6_H_7_N^+^, singly ionized aniline molecule) and at m/z = 40 amu (Ar^+^). In accordance with what was published in^20^, positive ions with a higher mass than the precursor can be measured.

### Formation of negative ions

In principle there are several different pathways leading to the formation of negative ions in low temperature plasmas. The article of E. Stoffels gives a good overview of the different production and destruction mechanisms of negative ions in low pressure discharges^58^ Although volume production via resonant electron attachment is the most common channel for the production of negative ions^39,41,50,69–71^, it has been shown that surface reactions can be an important source of negative ions as well^40,72,73^. For example the formation of H^−^ or D^−^ ions on the surface of different materials was highlighted for hydrogen plasmas^74–80^, and it is known that O^−^ ions can be created on target materials during reactive magnetron sputtering^81–84^. Several production channels of negative ions based on surface reactions are possible. The contribution of (high energy) positive ions is usually involved, for example through the sputtering of an adsorbed atom or molecule by a positive ion^85–87^, leading to the formation of a negative ion, or by backscattering of an incoming positive ion as a negative ion^85–87^. It was furthermore demonstrated that the collision of a positive ion with a neutral species within the sheath can lead to the formation of a positive-negative ion pair^72^.

**Figure 3:**
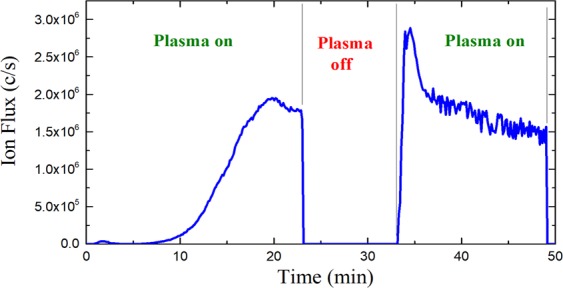
Temporal evolution of the negative ion flux entering the mass spectrometer (sampling orifice – plasma = 0 cm) for m/z = 90 amu (dominant negative ions). At t = 0 min the vacuum chamber is clean. The experiment was performed with a pulsed discharge (pulsing frequency = 200 Hz) at 0.18 mbar (argon: aniline = 20: 1 sccm) and with an input power of 5 W.

Experiments performed in this study indicate that in the present case of a low pressure aniline plasma, surface reactions may play an important role in the production of negative ions. Figure 3 shows the temporal evolution of the signal of the most prominent negative ions (m/z = 90 amu) measured by means of mass spectroscopy. In this experiment the mass spectrometer is positioned at a plasma – sampling orifice distance of 0 cm (see Fig. 7(b)). The experiment was started with electrodes that had been cleaned according to the procedure described in section 2 (“Experimental set-up”). Figure 3 shows that within the first minutes after the ignition of the plasma, no negative ions could be measured. Only after about 7 minutes, the negative ion signal started to increase slowly to reach a maximum value about 20 minutes after the discharge was switched on. The plasma was subsequently switched off for 10 minutes. When the discharge was switched on again, the negative ions appeared much faster, reaching a maximum value in less than 1 minute. This behavior is highly reproducible: each time the discharge is ignited in a clean chamber with clean electrodes it takes several minutes for the negative ions to appear. If however the discharge is ignited in a chamber in which an aniline plasma has been running before, the negative ions appear much faster - even if the chamber has been pumped down or vented in the meantime. The same experiment performed with other negative species (m/z = 26, 53, 80, 117, 144 and 208 amu) lead to a similar result. This indicates that the presence of a thin polyaniline layer on the electrodes is a sine-qua-non for the production of negative ions in the current case. The initial deposition of a thin layer of plasma synthesized polyaniline (pPANI) on the electrodes is occurring within the first minutes after plasma ignition, resulting in different plasma-surface interactions with the electrodes and leading eventually to the formation of negative ions.

**Figure 4:**
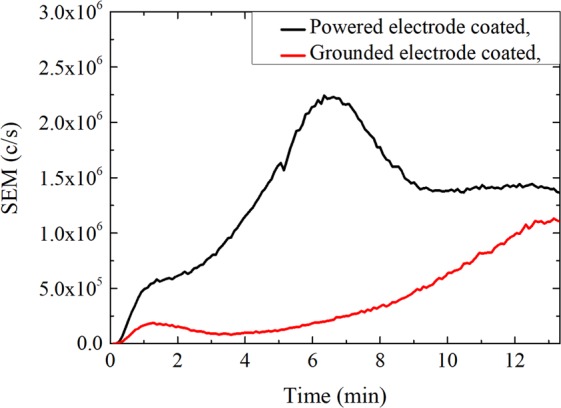
Temporal evolution of the negative ion flux entering the mass spectrometer (sampling orifice – plasma = 0 cm) for m/z = 90 amu (dominant negative ions). At t = 0 min the vacuum chamber is clean and one of the two electrodes is coated with a thin layer (a few tens of nanometers) of pPANI. The experiments were performed with pulsed discharges (pulsing frequency = 200 Hz) at 0.18 mbar (argon: aniline = 20: 1 sccm) and with an input power of 5 W.

In order to understand better the nature of surface reactions on the electrodes, a similar experiment was performed with only one of the two electrodes coated at a time by a thin layer of pPANI (see Fig. 4). The results show: if either the powered electrode alone or the grounded electrode alone is coated with pPANI, the negative ions are produced with a short delay after plasma ignition (less than one minute), but in a smaller amount in comparison with the situation were both electrode are coated. A rather long increase (6 to 12 min) of negative ion signal after the plasma was switched on was however still measured in both cases and may be due to the deposition of pPANI on the uncoated electrode. The fact that the negative ion signal is higher when the powered electrode is coated with pPANI might indicate that positive ions are involved in surface reactions on the electrodes leading eventually to the formation of negative ions. Indeed, the presence of a self bias on the powered electrode induces a stronger positive ion bombardment on its surface.

As presented in Fig. 2(b), the two dominant positive ions measured in this discharge are Ar^+^ (m/z = 40 amu) and C_6_H_7_N^+^ (m/z = 93 amu, singly ionized aniline molecule). In order to isolate the influence of argon ions on surface reactions, a pure argon plasma was ignited under similar experimental conditions (argon flow = 20sccm, process pressure = 0.08–0.3 mbar) and with both electrodes coated by a thin layer of pPANI. The coating was performed using a pulsed plasma (f = 25 Hz) ignited in a mixture of argon and aniline (20/1sccm) at 0.18 mbar and with 5 W input power. The deposition duration was set to 15 min and the coating thickness was 80 nm on the grounded electrode and 150 nm on the powered electrode. As a result, with powers ranging from 5 W to 50 W and with pulsing frequencies ranging from 25 Hz to 1200 Hz no negative ions could be measured when the discharges were operated in pure argon (plasma – sampling orifice = 0 cm). This indicates that the sputtering of the pPANI film on the electrodes by Ar^+^ ions is not sufficient to lead to the formation of negative ions. In addition, if the proportion of aniline with respect to argon is gradually decreased by lowering the aniline gas flow while keeping a constant total pressure inside the vacuum chamber, the density of negative ions measured by mass spectroscopy decreases and eventually reaches zero. The presence of some specific species, for example C_6_H_7_N^+^ ions, other gaseous species such as the aniline molecule, or specific radicals/ions is obviously necessary for the production of negative ions in the Ar/Aniline plasmas studied.

**Figure 5:**
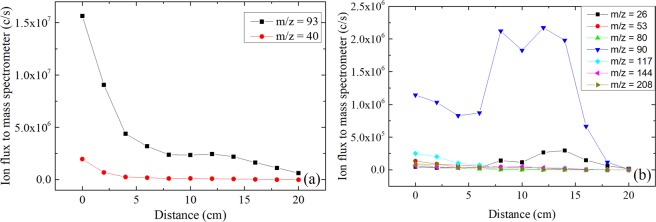
Flux of the main positive ions (**a**) and negative ions (**b**) entering the mass spectrometer for different plasma – sampling orifice distances. Experiments were performed with a pulsed discharge (pulsing frequency = 200 Hz) at 0.18 mbar (argon: aniline = 20: 1 sccm) with 5 W input power.

In a next step, negative ions were measured by means of mass spectroscopy but this time with different plasma – sampling orifice distances, ranging from 0 to 20 cm (thanks to the movable Hiden EQP 1000 mass spectrometer system). As expected, the relative intensity of positive ions signal decreases with increasing distances from the discharge (see Fig. 5(a)), and this behavior is similar for most negative ions (see Fig. 5(b)). But surprisingly, for m/z = 26, 90 and 144 amu, the signal of negative ions increases significantly for plasma – sampling orifice distances ranging from 8 cm to 14 cm (see Fig. 5(b)). This result may indicate a different production channel (as e.g. attachment of low energy electron) for these species compared to other negative ions (for which m/z = 53, 80, 117 and 208 amu). Under certain experimental conditions, for example when a gas mixture with a higher proportion of aniline is used to ignite the plasma (leading to a higher electronegativity), a similar effect (i.e. an increase of ions density with increasing distances between the mass spectrometer and the plasma) is also noticeable for positive ions for which m/z = 40 amu (Ar^+^) and 93 amu (singly ionized aniline). With increasing distances from the electrodes i.e. in the spatial afterglow region of the discharge, both the electron density and the electron temperature are expected to decrease continuously. Consequently the production of positive ions is expected to decrease with the increase of the plasma – sampling orifice distance. However, the behavior of electronegative plasmas is known to be extremely complex, especially in the boundary region of the discharge. Because of the specific design of the vacuum chamber (electrode edge to wall distance = 24 cm) the (grounded) mass spectrometer sampling orifice is much nearer to the plasma than the walls of the chamber. It can be expected therefore that the presence of the spectrometer will influence the measurements as it modifies the electric field lines inside the chamber. Nevertheless, when a pure argon plasma was ignited under similar experimental conditions, all positive ions exhibited a constant decay of their signal along with the increase of the plasma - sampling orifice distance, confirming that the electronegativity of the plasma is involved in this behavior.

### Influence of negative ions on thin film deposition

The experiments presented in Fig. 5 were accompanied by deposition experiments. In these experiments, a silicon wafer was fixed on the mass spectrometer next to the sampling orifice which is as before located at a specific but variable distance from the plasma (see Fig. 1(b)). Thin film deposition was then performed for plasma – sampling orifice distances of 5, 10 and 20 cm. A similar batch of samples was produced under similar experimental conditions but using a continuous wave (CW) plasma. Because negative ions are constantly trapped inside CW plasmas, in contrast to pulsed discharges, it is possible to produce reference samples which were not exposed to negative ions during the film growth. For both types of plasmas, the deposition time was set to 10 minutes.

**Figure 6:**
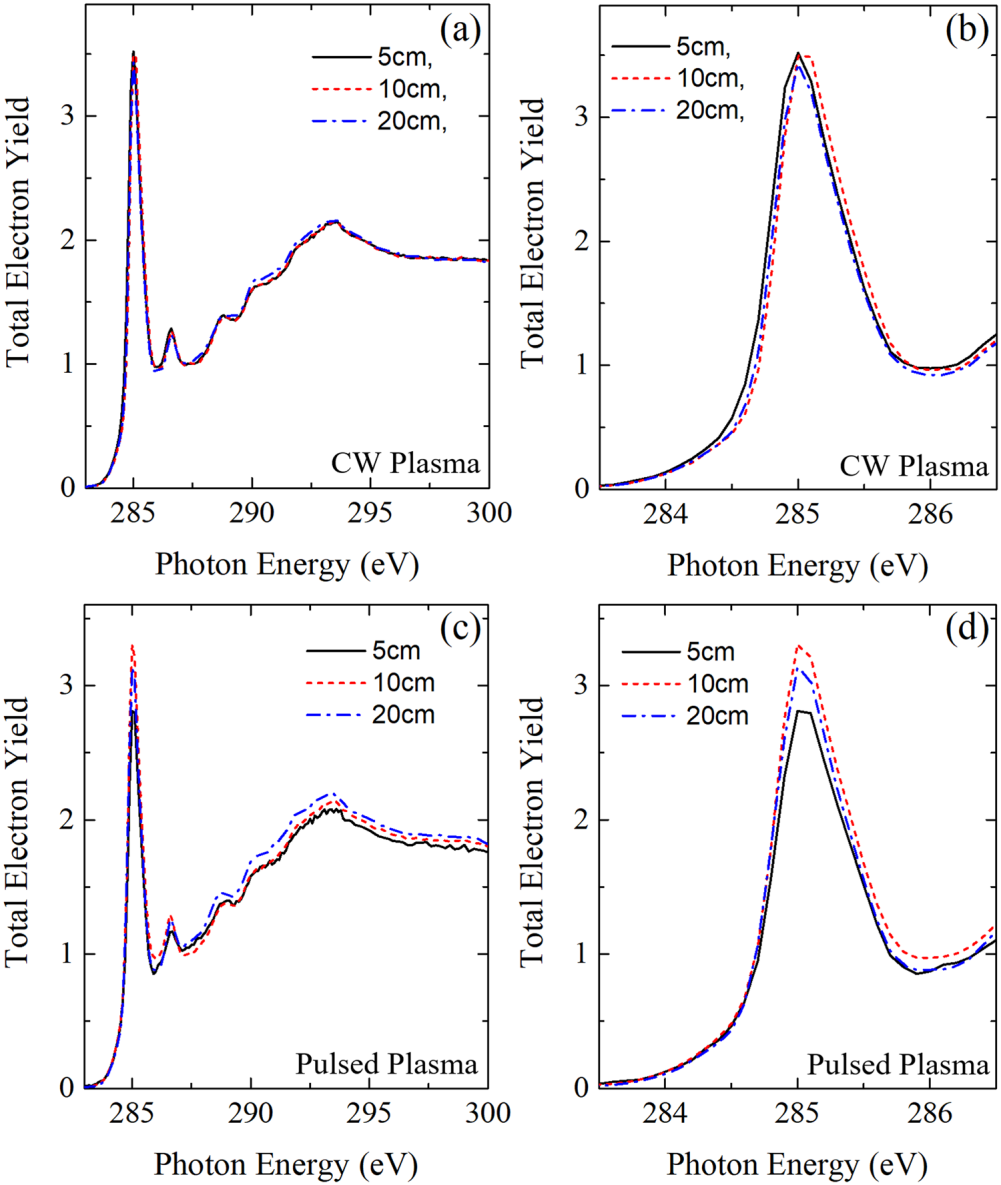
NEXAFS C K Edge measurements of pPANI deposited at different locations in the vacuum chamber for a continuous wave (CW) plasma (**a**)/(**b**) and a pulsed plasma (**c**)/(**d**) with a pulsing frequency of 200 Hz. For both measurements, experiments were performed with 5 W input power at 0.18 mbar (argon: aniline = 20: 1 sccm). All spectra are normalized at the photon energy value of 320 eV.

The samples were subsequently analyzed by means of NEXAFS spectroscopy (Fig. 6(c,d)). The measurements revealed for the C K Edge slight but significant differences in the deposited polymer structure depending on the position of the silicon sample in the case of pulsed plasmas. The main differences appear in the region between 284.9 and 285.4 eV (π* resonance of C=C bonding, which is correlated with varying amounts of aromatic units in the polymer structure). This peak reaches a maximum when the thin film is deposited in the zone where the negative ion density is the highest (i.e. plasma – sampling orifice = 10 cm). In comparison, for CW plasmas (Fig. 6(a,b)) the differences in NEXAFS spectra are much less pronounced, and this peak is just somewhat decreasing as the distance between the mass spectrometer and the plasma is increasing. The N K Edge and the O K Edge spectra of these materials did not exhibit significant differences depending on the position of the samples for both types of plasma.

This different behavior raises the question how far negative ions have an impact on the deposition of thin films in pulsed discharges where they can reach the substrate during plasma off phases. However, comparing thin films produced in a CW plasma (negative ions confined) and in a pulsed discharge (negative ions only intermittently confined) is certainly not conclusive enough since, besides the confinement of negative ions, CW and pulsed plasmas behave very differently in many points, even under similar experimental conditions. One prominent example concerns the formation of nanoparticles in reactive plasmas which can be avoided –under certain circumstances- by using pulsed discharges^20,47^. Another important issue concerns the degree of fragmentation of the initial monomer which is also influenced by the pulse of the plasma. Despite these concerns the results at least suggests that the negative ions might contribute to the thin film growth in the present case.

## Conclusion

The formation of negative ions was proved to occur in low pressure RF capacitively coupled plasmas operated in a mixture of argon and aniline vapor. The experimental results show a great variety of negative species with a dominant negative ion found for a mass to charge ratio of 90 amu, and the formation of heavy negative ions reaching more than two times the mass of the aniline precursor. The initial deposition of a thin layer of pPANI on the electrodes appears to be a necessary condition for the production of negative ions in this type of plasma. Surface reactions on both electrodes probably involving positive ions are an essential part of the (probably multi-step) mechanisms leading to the formation of negative ions.

In contrast to the positive ions and most negative ions whose density is decreasing with increasing distance between the plasma and the mass spectrometer sampling orifice, the negative ions with m/z = 26, 90 and 144 amu show a different behavior. Their signal shows a maximum value when the distance between the spectrometer and the plasma is increased to a certain range. This indicates a possible different production channel for these ions compared to other negative species created in this plasma. The attachment of low energy electrons to neutral species created by the plasma might be the reason for this phenomenon.

Deposition experiments in pulsed and CW discharges performed at different distances from the plasma suggest that the negative ions might contribute to the film growth. However, this contribution needs to be studied in more details.

## Methods

**Figure 7:**
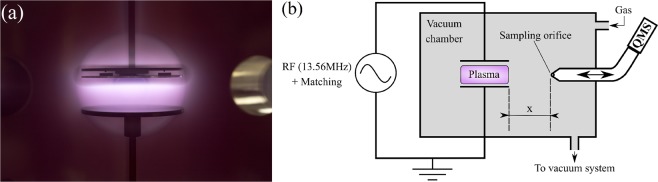
(**a**) Picture of the capacitively coupled plasma (the upper electrode is connected to the RF generator, whereas the bottom electrode is grounded). (**b**) Scheme of the mass spectroscopy experimental set-up. The quadrupole mass spectrometer (QMS) is here movable. The distance x corresponds to the distance between the electrode edge and the spectrometer sampling orifice.

RF capacitively coupled plasmas (CCP, f = 13.56 MHz) were ignited at low pressure in a vacuum chamber between two cylindrical parallel plane electrodes with a diameter of 12 cm and separated by 5.5 cm (see Fig. 7(a)). Experiments were performed with plasmas created in a mixture of aniline vapor (1sccm) and argon (20sccm) at different pressures ranging from 0.08 mbar to 0.3 mbar. The power applied to the electrodes was fixed to 5 W, and plasmas were either pulsed with a duty cycle of 50%, with pulsing frequencies ranging from 25 Hz to 1200 Hz, or driven in continuous wave (CW). Aniline vapor was generated by heating liquid aniline (Sigma Aldrich 242284) with a Nanosource device from Omicron Technologies, which provides a stable, modular gas flow to the vacuum chamber with a high reproducibility. The flow (mostly 1 sccm in this study) was calculated based on the manufacturer data and a batch of tests performed with the current set-up.

Mass spectroscopy measurements were carried out using a HIDEN EQP 1000 quadrupole mass spectrometer (QMS) for the analysis of positive and negative ions (see Fig. 7(b)). pPANI thin-films were deposited on intrinsic silicon, and material diagnostics (NEXAFS) were carried out at the beam line HE-SGM of the BESSY II Synchrotron facility in Helmholtz-Zentrum Berlin.

In order to ensure reproducible measurement, electrodes were cleaned between each experiment with an oxygen plasma (50 W, 3 hours) followed by an argon plasma (50 W, 1 hour).

## Data Availability

All data generated or analyzed during this study are included in this published article.
